# Dorsal Root Ganglion Stimulation in Patients With Refractory Abdominal Pain: A Case Series

**DOI:** 10.7759/cureus.83218

**Published:** 2025-04-29

**Authors:** Zeshan R Ali, Beker Karadaghy, Iqbal Khan, Geoffrey D Panjeton, Hess Panjeton

**Affiliations:** 1 Diagnostic Radiology, Saint Louis University School of Medicine, Saint Louis, USA; 2 Anesthesiology, Saint Louis University School of Medicine, Saint Louis, USA; 3 Anesthesiology and Critical Care, Saint Louis University School of Medicine, Saint Louis, USA; 4 Anesthesiology, Washington University School of Medicine, Saint Louis, USA

**Keywords:** dorsal root ganglion stimulation, neuromodulatory therapy, refractory abdominal pain, right upper quadrant abdominal pain, treatment of chronic pancreatitis

## Abstract

This case series highlights the use of dorsal root ganglion (DRG) stimulation for refractory chronic abdominal pain in three patients who failed to respond to conventional therapies. DRG stimulation modulates pain signals by targeting the sensory neurons present in the DRG. The three cases involved patients with chronic abdominal pain resulting from pancreatitis, endometriosis, and pancreatic cancer. DRG stimulation resulted in pain reductions of 100%, 90%, and 80%, respectively, accompanied by significant improvements in daily functionality and reduced analgesic use. These findings underscore the potential of DRG stimulation as a targeted, effective treatment for refractory abdominal pain. Further research with larger cohorts is warranted to validate these results and refine treatment protocols.

## Introduction

Chronic abdominal pain is a debilitating condition associated with significant declines in quality of life. When unresponsive to conventional therapies such as physical rehabilitation, pharmacologic analgesics, and sympathetic plexus nerve blocks, it poses a clinical challenge for both patients and healthcare providers. Standard management approaches, including opioids, nonsteroidal anti-inflammatory drugs (NSAIDs), anticonvulsants, nerve blocks, and epidural injections, may provide only temporary relief, have systemic side effects, or fail to adequately control refractory pain. Furthermore, psychosocial factors such as anxiety and depression often compound the burden of chronic pain, making effective treatment even more challenging [1]. Emerging patient-specific interventions, such as neuromodulation, have shown promise in addressing pain that is resistant to standard treatments [2].

Dorsal root ganglion (DRG) stimulation is a neuromodulatory therapy that received FDA approval in 2016 for chronic pain management [2,3]. This approach directly modulates nociceptive signals from peripheral organs by targeting sensory neurons at the DRG [2]. Compared to spinal cord stimulation (SCS), DRG stimulation provides more precise targeting, making it advantageous for focal pain syndromes [2,4]. However, its application for chronic abdominal pain remains underreported despite its potential benefits [5]. This case series explores the use of DRG stimulation in three patients with chronic abdominal pain unresponsive to conservative measures. These cases further contribute to the growing literature highlighting DRG stimulation as a targeted approach for managing refractory pain and improving quality of life.

## Case presentation

Table 1 provides an overview of the demographic and clinical characteristics of three patients treated with DRG stimulation for chronic abdominal pain of varying etiologies, including chronic pancreatitis, postoperative adhesions, endometriosis, and pancreatic cancer. Reported variables include patient age, sex, diagnosis, prior treatment modalities, DRG lead placement, pain reduction following implantation, and functional outcomes.

**Table 1:** Clinical characteristics and outcomes of patients treated with dorsal root ganglion (DRG) stimulation for chronic abdominal pain. NSAIDs: nonsteroidal anti-inflammatory drugs; QoL: quality of life; DRG: dorsal root ganglion

Case	Age/Sex	Diagnosis	Previous Treatments	DRG Lead Placement	Pain Reduction (%)	Outcome
1	36/F	Refractory lower abdominal pain secondary to pancreatitis, with adhesions and endometriosis	NSAIDs, chiropractic therapy, celiac/hypogastric plexus blocks, opioids, gabapentin	T12, L1 (left)	100	Complete relief, reduced meds, improved function
2	43/F	Chronic right upper quadrant pain due to chronic pancreatitis	Analgesic medication management	T9, T10 (right)	90	Improved activity and QoL, some discomfort from friction
3	55/F	Chronic left upper quadrant pain due to pancreatic cancer	Medications, sympathetic plexus nerve blocks	T10 (left)	80	Return to functional baseline, improved mobility, reduced analgesics

**Figure 1:**
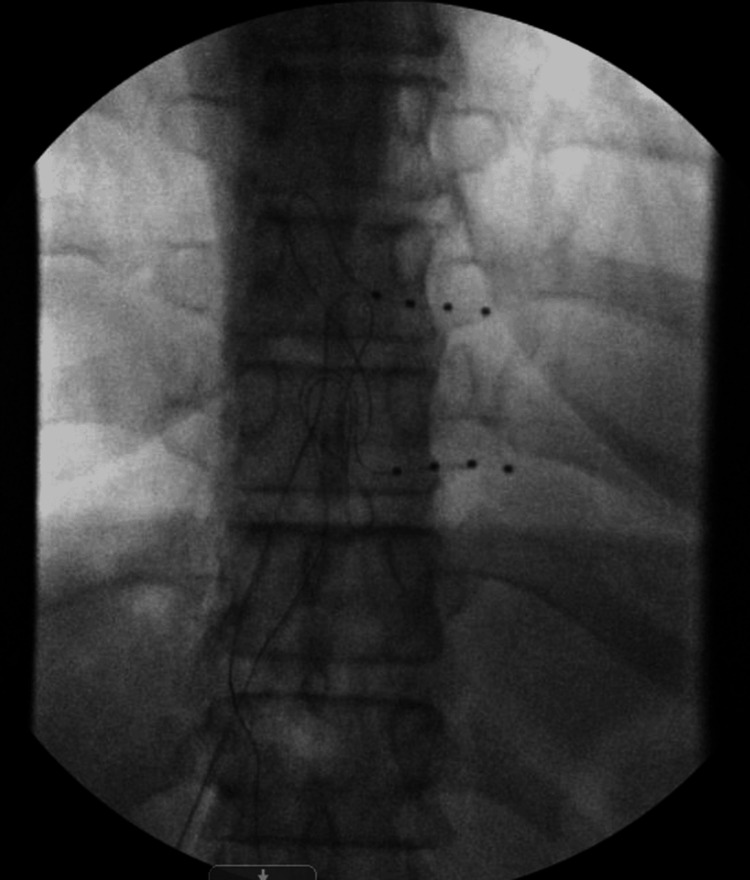
Intraoperative fluoroscopic image (anteroposterior view) showing dorsal root ganglion stimulation leads placed at the T9 and T10 levels on the left side (Case 1). Lead positioning was confirmed via fluoroscopy during the trial phase.

Figure 1 illustrates the intraoperative fluoroscopic image from Case 1, depicting the placement of DRG stimulation leads at the T9 and T10 levels on the left side.

## Discussion

This case series underscores the potential effectiveness of DRG stimulation for patients with chronic abdominal pain that has not responded to conventional treatments. By targeting specific sensory neurons, DRG stimulation offers focused pain relief, which may present an advantage over broader neuromodulation methods like SCS, which can impact a larger, less specific area [4].

The reported pain reductions of 100%, 90%, and 80% in the three patients align with existing studies on DRG stimulation for neuropathic pain. Notably, prior research indicates that approximately 63% of patients undergoing DRG stimulation experience a greater than 50% reduction in pain [2,4]. Beyond pain relief, all three patients experienced notable improvements in daily functioning and reduced dependency on pain medications. This is especially important in the context of the ongoing opioid crisis, reinforcing the importance of finding effective alternatives to opioid use for chronic pain management [6]. These results strengthen the case for DRG stimulation as a viable option for chronic abdominal pain that has failed to improve with other treatments.

One key advantage of DRG stimulation is its ability to target specific pain regions, reducing the need for systemic medications and their associated side effects [2,3]. Compared to interventions like celiac plexus blocks, which may require frequent repeat procedures, or intrathecal drug delivery, which introduces the risk of complications from catheter use, DRG stimulation presents a long-term, minimally invasive solution [6]. However, the precision of lead placement is crucial to treatment success, and individual anatomical variations could influence outcomes [4]. Additionally, while the short-term results are promising, the long-term durability of pain relief remains uncertain and warrants further investigation [9].

Psychosocial factors, such as depression and anxiety, are frequently observed in patients with chronic pain and may significantly impact treatment response [7]. Integrating psychological support alongside neuromodulation therapy could optimize patient outcomes and enhance the efficacy of DRG stimulation [7]. Furthermore, refining patient selection criteria is essential to ensure that DRG stimulation is used for those patients most likely to benefit from it [8].

A major limitation of this case series is the small sample size, which limits the generalizability of the findings. Future research should aim to include a larger cohort of patients with longer follow-up periods to confirm these results. Additionally, studies should explore potential complications, refine lead placement techniques, and develop standardized protocols for patient selection and outcome optimization.

## Conclusions

DRG stimulation is a promising intervention for refractory chronic abdominal pain, offering significant pain relief and improved functionality in this case series. These findings add to the growing evidence supporting DRG stimulation as a targeted treatment modality for conditions beyond its traditional indications. Further research is essential to confirm these benefits, establish standardized protocols, and optimize patient outcomes.
